# Local Solid Shape

**DOI:** 10.1177/2041669515604063

**Published:** 2015-10-30

**Authors:** Jan Koenderink, Andrea van Doorn, Johan Wagemans

**Affiliations:** University of Leuven (KU Leuven), Belgium; Faculteit Sociale Wetenschappen, Universiteit Utrecht, The Netherlands; Faculteit Sociale Wetenschappen, Universiteit Utrecht, The Netherlands; University of Leuven (KU Leuven), Belgium

**Keywords:** shape index, Casorati curvature, solid shape, surface curvature

## Abstract

Local solid shape applies to the surface curvature of small surface patches—essentially regions of approximately constant curvatures—of volumetric objects that are smooth volumetric regions in Euclidean 3-space. This should be distinguished from local shape in pictorial space. The difference is categorical. Although local solid shape has naturally been explored in haptics, results in vision are not forthcoming. We describe a simple experiment in which observers judge shape quality and magnitude of cinematographic presentations. Without prior training, observers readily use continuous shape index and Casorati curvature scales with reasonable resolution.

## Introduction

We use *solid shape* to indicate *volumetric shape* (Koenderink, 1990), in contradistinction to *pictorial shape* or *pictorial relief* (Hildebrand, 1893; Koenderink, van Doorn, & Kappers, 1992; Schlosberg, 1941). The key distinction is that pictorial relief has a distinguished direction from which it is seen, referred to as *depth* (Koenderink, van Doorn, & Wagemans, 2011), whereas solid shape can be seen from arbitrary directions. Relief is seen in pictures, hence *pictorial relief.* The generic example in vision science is the circular disk filled with a linear gradient of gray tone, which is often seen as a cup or cap (Metzger, 1975; Wagemans, van Doorn, & Koenderink, 2010). Solid shape is experienced when one walks around a sculpture or rotates a small object with the hands (Koenderink, 1990; Rogers, 1969). Solid shape is also experienced in cinematographic sequences.^1^ In the latter case, one does not actively change the viewing direction, but one is passively following the view of the cinematographer. The result is the same to the extent that there is no singular depth direction. Because the cinematographer may restrict the available viewing directions, a certain degree of—often intended—ambiguity remains.^2^

The Euclidean theory of local surface shape (see later) assumes that the three Cartesian dimensions are mutually equivalent. This equivalence fails in pictorial space. As a consequence, the theory of local space of pictorial relief is different from that in Euclidean space. One consequence is that it makes little sense to use a continuous shape index scale for pictorial relief. The obvious alternative is to use a categorical scale based on the signs of the principal curvatures (in terms of the differential geometry of pictorial space!). Observers easily use such a scale (Dövencioğlu, Wijntjes, Ben-Shahar, & Doerschner, 2015; Koenderink, van Doorn, & Wagemans, 2014). If one asks whether observers are able to work with a continuous scale, one has to do experiments in Euclidean space. Here, we report on such an attempt.

The formal theory of smooth shapes on a local scale is differential geometry (Hilbert & Cohn-Vossen, 1932; Koenderink, 1990). “Local” means that one studies the lowest order deviations from planarity, generically that is the second order, the so-called curvature of the surface (Gauss, 1827). Thus, “local” is a technical, formal concept. It simply means that one uses no derivatives of order higher than two in the differential geometry. For the case of pictorial relief, the deviations are necessarily in the depth direction, whereas the structure of the depth dimension differs qualitatively from those that span the picture plane. For the case of solid shape, one naturally measures the deviation from the local tangent plane in the direction of the local normal.^3^ Here, all three dimensions are equivalent. This case has been studied in haptics (Kappers, Koenderink, & Lichtenegger, 1994; Kappers, Koenderink, & te Pas, 1994). The cases of pictorial relief and solid shape are categorically distinct. In previous experiments, we have only regarded *pictorial* shape. In this experiment, we regard *solid* shape in a controlled, cinematographic setting.

### Formal Theory of Local Solid Shape

One can always establish a Cartesian coordinate system {*x*,*y*,*z*}, where the *z*-coordinate is in the normal direction and the *xy*-coordinates are in the tangent plane, such that the normal deviation from the tangent plane can be expressed as

$$z(x,y)=\frac{1}{2}(k_{1}x^{2}+k_{2}y^{2})+{\text{o}}^{3}[x,y]$$

such that *k*_1_* ≥ k*_2_. The *k*_1,2_ are the so-called “principal curvatures” of the surface at the origin (Euler, 1767; Meusnier, 1776). The approximation ignores cubic and higher order terms. The latter may actually become dominant at some distance from the origin, thus the description is properly called “local.” This is the meaning of “local” throughout this article.

For a sphere of radius *R*, one has *k*_1,2 _= 1/*R*. Thus, the curvatures are reciprocal radii and are of dimension [length]^−1^. The ratio *k*_1_/*k*_2_ measures the anisotropy of the curvature. When one of the principal curvatures is zero, one has the case of a cylinder; when the curvatures are in different senses (as indicated by sign^4^), one has a saddle surface. When both curvatures are zero, one obtains the planar case, that is to say, the normal deviation is dominated by the cubic terms. Of course, the planar case is singular, occurring with probability zero.^5^ We will ignore it in this study. Formally, the plane has no shape in this formalism, analogously to white and black lacking a hue in the context of color.

Whereas the ratio of principal curvatures is a dimensionless number correlated with what might be called the “quality” or “shape proper,” one also desires a measure of magnitude. A convenient measure is the variance or standard deviation (properly defined) of the normal deviation.^6^ Both the quality and magnitude measures can be conveniently defined so as to satisfy some a priori desirable constraints.

**Figure 1.**
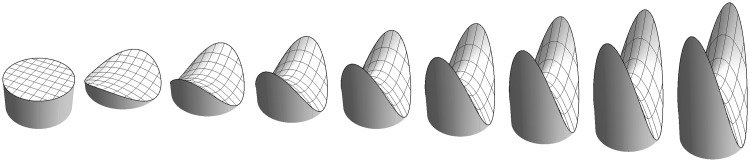
A symmetrical saddle at various Casorati curvatures. The leftmost instance has curvature zero, thus is planar. For the planar case, the shape index is undefined; thus, this is not really a “saddle of zero Casorati curvature,” it is a mere “flat.” One might as well say it to be a “cap of zero Casorati curvature!” Thus, the planar case is not really a shape, just like “black” does not own a proper hue. In the real world “nothing” is the same as “anything” if you allow infinite discriminatory power! In the same sense, flat also means “no shape” as much as “any shape” (see also Koenderink et al., 2014).

The magnitude should perhaps be defined such as to be 1/*R* for a sphere of radius *R*. The standard deviation is indeed such a measure (Koenderink & van Doorn, 1992)

$$c=\sqrt{\frac{k_{1}^{2}+k_{2}^{2}}{2}}$$

the so-called Casorati curvature (Casorati, 1970). It is a non-negative magnitude of dimension [length]^−1^ that equals 1/*R* for a sphere of radius *R*. See Figure 1. The definition of the Casorati curvature as the standard deviation of the distances from the tangent plane is novel and will not be found in textbooks of differential geometry. It seems a particularly apt interpretation in the context of perception.

**Figure 2.**
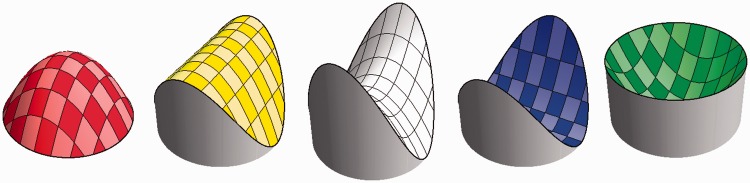
A cap, ridge, saddle, rut, and cup. The shape indices are, respectively, −π/2, −π/4, 0, +π/4, +π/2. These have the same Casorati curvature. The colors are those of the hue scale used in this article. Notice that complementary colors denote complementary—related as mold and cast—shapes. Thus, the cap fits the cup, the ridge the rut, whereas the saddle fits itself. The scale is actually continuous, thus—for example—there is a smooth range between cap and ridge. The symmetrical caps and cups are special, technically known as “umbilical.” Likewise, the saddle shown here is special, the “symmetrical saddle” or “minimal surface.” The ridge and ruts are mere transition points. However, when coarse graining, they also subtend finite (though fuzzy) ranges.

A quality measure is more involved. It should evidently be a dimensionless number. However, the ratio has the drawback that it is invariant with respect to a simultaneous sign change of both principal curvatures. Such an operation inverts the z-coordinate, thus it transforms a form into its *negative*, related to the original like the mold to the cast. An example is the inside and outside of an egg shell. Notice that the symmetrical saddle is congruent to its own mold! Thus, it would be natural that it had measure zero (because +0 equals −0). Another useful observation is that the case of equal principal curvatures is very special, and you cannot get any “rounder” than that. Thus, a quality measure should be defined on a finite, symmetrical segment like [−*α*,+*α*] (with constant *α *> 0), where zero corresponds to the symmetric saddle and the endpoints to the inside and outside of spherical shells. An example of such a measure is (Koenderink & van Doorn, 1992)

$$s=\arctan(\frac{k_{2}+k_{1}}{k_{2}-k_{1}})$$

which denotes the *shape index*. It evidently varies on the segment [−π/2,+π/2]. See Figure 2.

Now we can write

$$z(x,y;c,s)=c((\frac{x^{2}+y^{2}}{2})\sin s+(\frac{x^{2}-y^{2}}{2})\cos s)$$

which reveals the shape as a scaled mixture of a sphere with a symmetrical saddle. The Casorati curvature yields the overall scaling factor, whereas the shape index determines the mixing ratio.

### The Basic Shape Categories

The categorically distinct shapes can be characterized as follows (Figure 2):

*s *>* *+* *π/4 concavities (“like the inside of egg shells”)
*s*_ _= +π/4 concave cylinder (“like the inside of reeds”)
−π/4* *<* s *<* *+* *π/4 saddle shapes (“like a horse’s saddle”)
*s*_ _= −π/4 convex cylinder (“like columns”)
*s *<* *−π/4 convexity (“like the outside of egg shells”)

Here, the quoted descriptions are from Alberti’s (1435/1972) taxonomy, except for the saddles, which were not mentioned by Alberti.

The first taxonomy that includes saddles was due to Gauss (1827). For centuries neither artists nor scientists noticed that Alberti’s taxonomy was incomplete. Yet, it is possible to show that 57% of the points of a Gaussian random surface have a saddle shape (Koenderink & van Doorn, 2003; Lillholm & Griffin, 2009). Alberti (1435) included the planar case (“like a water surface”) in his taxonomy. Gauss was wise to ignore it as a singular case.

### The Aim of the Current Research

The categorical scale is all one can use in cases where only the signs of the principal curvatures are available. This frequently happens in vision, due to unknown foreshortening effects. The full continuous scale is expected to become available only in true cases of *solid shape perception*. We are interested to establish whether human observers are able to use the shape index and Casorati curvature scales in the latter case and whether the saddle region is in any way special. These questions have not been addressed by previous research, as will be discussed next.

### Relations to Previous Work

An early study using the shape index scale is Van Damme and van de Grind (1993). These authors used head movement induced motion parallax. Since the human visual system appears to use only velocity, not acceleration (Koenderink & van Doorn, 1975), this would yield an affine range scale (Koenderink & van Doorn, 1991). Observers were able to use the categorical scale with moderate precision, responses being roughly independent of the Casorati curvature.

Another early study is de Vries, Kappers, and Koenderink (1993). These authors used binocular disparity to define reliefs. Observers used the categorical scale with moderate precision. The precision increased with the Casorati curvature, but the means were independent of it.

In a follow-up study (de Vries, Kappers, & Koenderink, 1994), it was shown that observers readily tolerate different spatial attitudes (non-frontal presentation). There were strong indications that thresholds and variances are high for saddle shapes. Cylindrical shapes (either ruts or ridges) were detected most easily and precisely.

Phillips and Todd (1996) did local shape index estimates on various locations of random Gaussian reliefs presented stereoscopically. They found evidence that precision increased with larger field size. Please notice that the term *local* as used by these authors is categorically different from our use in this article! There was again evidence for highest precision being obtained with cylindrical shapes. These authors presented computer simulation results showing that cylindrical shapes are most abundant on random surfaces (as proved formally by Koenderink & van Doorn, 2003).

Perotti, Todd, Lappin, and Phillips (1998) used motion parallax presentations. They showed that observers readily used the shape index scale but did not seem to be able to use the Casorati curvature scale. They used first-order flow perturbations, showing that observers based their judgments on second-order spatial derivatives of flow patterns.

In a recent study, Dövencioğlu et al. (2015) used the shape index to study shape from specular flow. This work focused primarily on shape from specular flow, shape index being used as a tool. Clearly, this study did not address the same issues about local solid shape as our study does.

Notice that all these previous studies focused on other aspects of vision than shape index or Casorati curvature per se and that most of them were not done in the context of isotropic Euclidean space. It remains unclear whether observers are actually able to use a continuous shape index scale instead of a categorical one and whether they are able to judge Casorati curvature at all. This is of some conceptual interest, although, in practice, most settings in vision will indicate the use of a categorical scale.

## Methods

### Observers

Participants were AD, female, aged 66; JK, male, aged 72; and JW, male, aged 51. The observers were well aware of the general theory of local shape. This is indeed required since otherwise *shape index* or *Casorati curvature* scales would make no sense to the observer. The observers did not have practice using these scales, although they had practice with the use of a categorical shape index scale in the setting of pictorial relief (Koenderink et al., 2014).

Observers viewed from a distance of 78* *cm. They used their preferred correction when necessary. All participants had normal corrected acuity. They used a chin-rest and monocular viewing.

The stimuli were presented on a DELL U2410f monitor, a 1920* *×* *1200 pixels (517* *×* *323* *mm) liquid crystal display screen, in a darkened room. We used the standard Apple settings for white point and gamma. The stimulus filled the width of the screen.

### Stimulus and the Geometrical Framework

**Figure 3.**
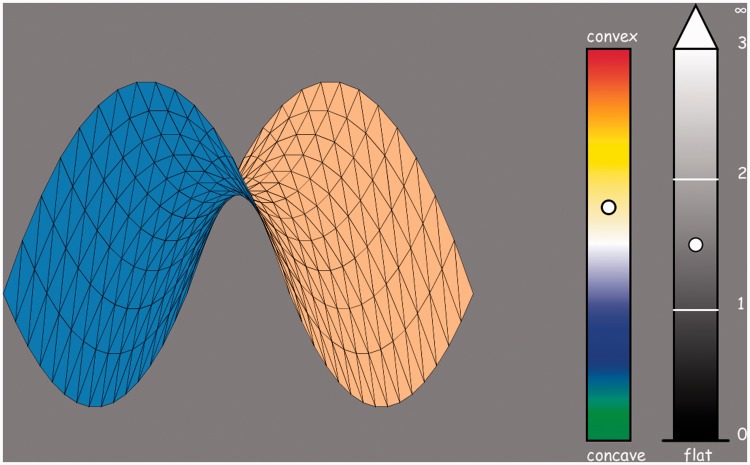
A frame from the screen presentation. This image fills the full screen. At left the surface, its top-side brownish, bottom-side bluish. At right the shape index scale, colored in the manner discussed in the text. It is a segment centered at the origin (white), so the bottom (green) and top (red) are the limits of the scale. The Casorati curvature scale is a ratio scale, the origin (planar patch) at the bottom. This scale is indicated as *open ended* from above. Only the bottom part of the scale is actually used by the observers, the “veridical settings” would all be lower or equal than one. Two animations of the rotating surface (stimulus) along with the corresponding values of the shape index and the Casorati curvature are available as Supplementary material.

The stimuli were computer graphics renderings of local shapes (meaning: no cubic and higher order terms) that were shown as rotating about the *z*-axis at uniform speed (Figure 3). Thus, the shape—as defined by the two principal curvatures—is revealed over time. During a full revolution, each principal curvature appears two times as the contour of the silhouette, thus as a planar curve in the screen. Observers were encouraged to judge shape and curvature magnitude on the basis of their (strong) three-dimensional *solid shape* impressions.

This setting differs from pure relief presentation by way of pictorial cues, motion parallax, or binocular disparity, as in the previous studies reviewed earlier, in that there is more information available. In principle, an algorithm would be able to yield perfect estimates of both shape index and Casorati curvature, as is not possible in case of the aforementioned presentations.

## Experiment

Adjacent to the movie of the rotating shapes, the display shows two scales, one for the shape index and one for the Casorati curvature (Figure 3). The observers have to drag cursors along these scales to the locations of their judgements. The response time was in no way restricted. When satisfied, they hit the spacebar of the keyboard, which then results in the next trial being displayed. Solid shapes of a variety of shape indices and Casorati curvatures were presented in randomized order.

A single session involved 121 trials, all combinations of 11 shape index values (−1.57, −1.26, −0.943, −0.628, −0.314, 0.00, 0.314, 0.628, 0.943, 1.26, 1.57), and 11 Casorati curvature values (0.083, 0.167, 0.250, 0.333, 0.417, 0.500, 0.583, 0.667, 0.750, 0.833, 0.917 times the reference curvature). Each observer completed three sessions with at least several hours in-between sessions.

The surface shown in the movie had different colors at its upper and lower side. This fixes the normal direction, which is necessary to be able to distinguish convexities from concavities, yet shows both upper and lower sides, which helps in cases one of the sides would become occluded.

The shape index scale is an absolute scale, but the Casorati curvature scale is a ratio scale for which observers need a unit reference. The reference “unit Casorati curvature” was shown to them before the first trial. They could also review this reference at any time by holding a certain key. The reference and the stimulus were never simultaneously visible though. As reference, we used a convex spherical surface (shape index −π/2).

The shape index scale was marked with a continuous color sequence. The colors were chosen in such a way that shape differing by sign only were given complementary hues. Of course, this implies that the symmetrical saddle was represented by the achromatic hue (white). Specifically, we used

convexity (*s*_ _= −π/2) red

ridge (*s*_ _= −π/4) yellow

symmetric saddle (*s*_ _= 0) white

rut (*s*_ _= +* *π/4) blue

concavity (*s*_ _= +* *π/2) green,

and the intermediate colors being obtained by linear interpolation in RGB-space.

The Casorati curvature scale should—in principle—range from zero to infinity, which is a practical impossibility. To avoid crowding effects at the top of the (displayed) scale, we made sure that the scale was rather too long. Moreover, we gave the observer the option to indicate judgements as outrunning the scale.

## Analysis

Observers considered the task an intuitive one and consequently response times were short: AD 8.0* *s (median, interquartile range 6.3–11.2* *s), JK 2.7* *s (2.3–3.1* *s), and JW 13.8* *s (10.8–19.6* *s). We did not find a dependence of response time upon the value of the shape index, so apparently saddles and caps or cups take roughly equal effort.

**Figure 4.**
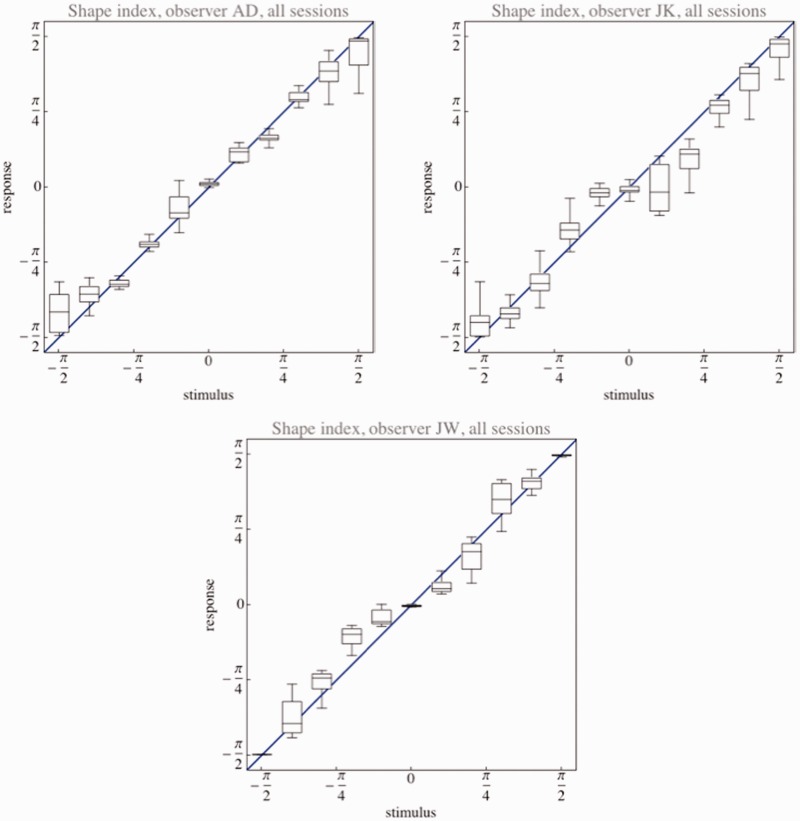
Shape index settings pooled over all sessions of single observers. The whisker box graphics indicates the range, the interquartile range, and the median. The blue line indicates the identity. The identity yields a satisfactory fit, though a slight cubic modulation (“S-curve” tendency) is perhaps suggested.

Observers use the full shape index scale (Figure 4); there are no indications that they employ a categorical scale and they seem to do about equally well in the saddle as compared with the cup or cap ranges. The relation is a linear one, though we find that adding a cubic term yields a just significantly better fit (*p*-values in the .01 range) for some of the individual sessions for each subject. This can also be (but only just) detected by inspection of the overall data. It may perhaps have to do with a certain reluctance to use the very limits of the scale.

**Figure 5.**
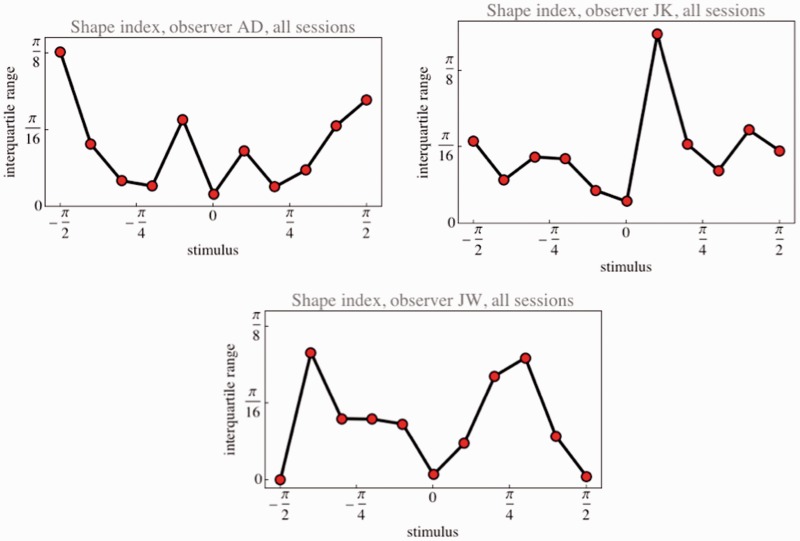
Interquartile ranges for the shape index settings pooled over all sessions of single observers. Notice that the functional dependencies are idiosyncratic.

The resolution, defined as the shape index range divided by the semi-interquartile range, is about 41 for AD, 34 for JK, and 43 for JW. Thus, observers discriminate at least ten times better than the mere categorical scale. The functional dependence of resolution on the shape index values fails to show a consistent pattern over observers (Figure 5). Perhaps one might be able to find one by averaging over many observers, but such a result would be of little interest, because essentially uninformative for any individual observer.

**Figure 6.**
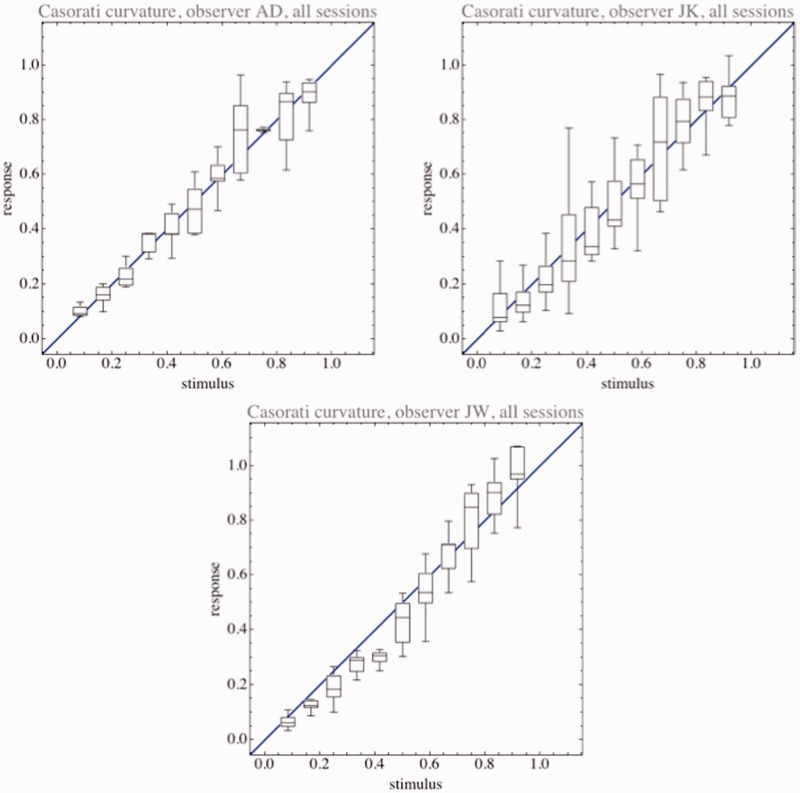
Casorati curvature settings pooled over all sessions of single observers. The whisker box graphics indicates the range, the interquartile range, and the median. To allow convenient comparison between observers, we normalized the best linear fit to the identity in all cases. The blue line indicates this identity.

**Figure 7.**
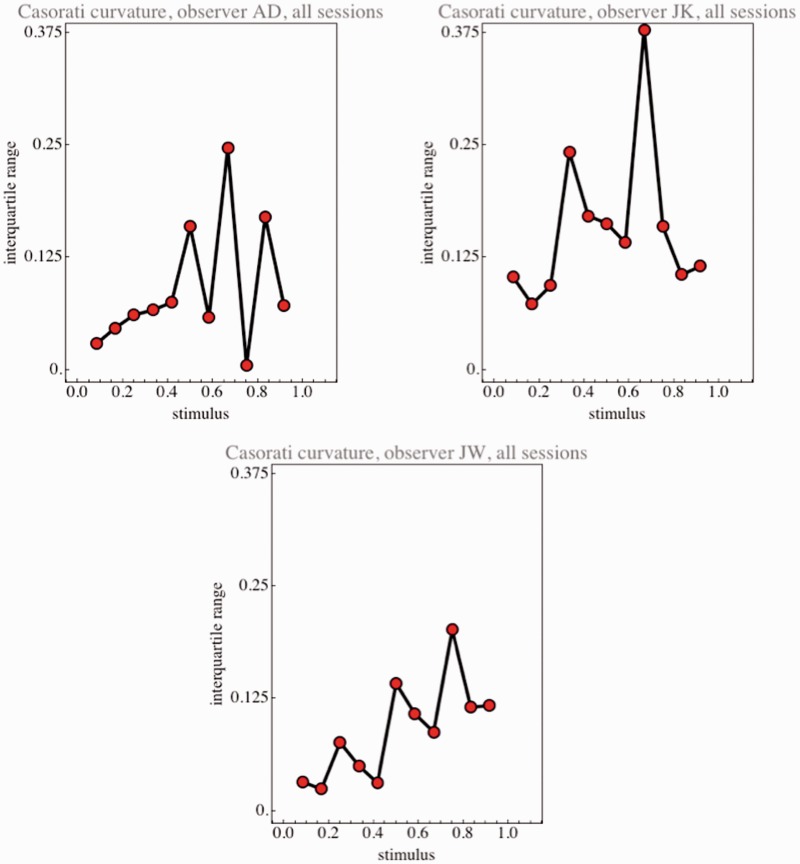
Interquartile ranges for the Casorati curvature settings pooled over all sessions of single observers. Notice that the functional dependencies are idiosyncratic, although one believes to spot a tendency—doing considerable coarse graining by eye—of a monotonic increase.

Observers were able to use the curvature scale too (Figure 6). This was more difficult because they had to keep the unit reference example in mind at all times. In a simultaneous comparison, it is likely that they would do much better. The results suggest a Weber fraction (Helmholtz, 1867) of about 10%, of course, with a considerable absolute plateau. This is at most twofold the discrimination threshold for planar ellipses. In view of the obviously large idiosyncratic spreads (Figure 7), there is little more that could be said.

## Discussion and Conclusions

The major results appear clear-cut. None of the three observers had any problems to use the shape index or the Casorati curvature scales. Indeed, they could do so without prior training, although they evidently understood the nature of the scales. Doubtless, novel, naive observers would certainly need some tuition on what the scales mean before starting on using them, as is true for essentially any use of scales.

There are no indications that the observers really interpolate on a categorical scale and we find no evidence that would serve to single out the saddle range as in any way special. The shape index scale resolution is much better than the coarse categorical scale. On the detailed level, the results show apparently idiosyncratic characteristics. These results allow us to draw some conclusions of general relevance.

First of all, the parameterization of local shape in terms of a quality—the shape index—and a magnitude—the Casorati curvature—is apparently a very natural one that observers are able to apply without preliminary training, given that they understand what the scales signify. Naive observers can be taught the scale by showing them examples, there being no pressing need to teach them formal differential geometry. This was to be expected in view of the fact that Alberti’s nominal scale *cup,* *rut,* *ridge,* and *cap* was generally accepted by scientists and artists for several centuries. However, it is perhaps remarkable that no one ever reported the saddle category as missing from this taxonomy.

The shape index seems to be the obvious “natural” parameterization of local shape as a quality. Indeed, there appears to be no other contenders. *Shape* as a quality has to be independent of both size and spatial attitude, so almost all that is left is the ratio of naturally sorted principal curvatures.

From our data, the saddle category is nothing special in the perception of solid shape. Observers use it naturally and precisely. This is not surprising in view of the ecological abundance of saddle shapes. On a random Gaussian surface, 57% of the local shapes is of the saddle category (Koenderink & van Doorn, 2003). It scores high in the Bayesian prior. Then why did nobody notice? We have suggested earlier (Koenderink et al., 2014) that this may be due to the fact that the cap category is naturally associated with solid bodies, which has again led to the general neglect of the remaining categories in the visual arts.

Observers discriminate much better—about an order of magnitude better—than categorically, say the Albertian categories augmented with the symmetric saddle (Koenderink et al., 2014). This has to be specific for the perception of solid shape as compared with pictorial shape. In the latter case, foreshortening precludes the quantitative comparison of curvatures in different directions, leaving one only with the coarse differentiation on the basis of mere sign of curvature. This reflects the essential difference between “real 3D” (or *space*) and 2* *+* *1D (visual field augmented with *depth*) pictorial space (Koenderink, van Doorn, & Wagemans, 2011).

Despite the good discrimination, it remains the case that one cannot name “in-between” instances. These have to be described in terms of the “anchors.” The cup, rut, saddle, ridge, and cap certainly appear prototypical instances, or anchors, very similar to the primary colors on the color circle.

Observers are also able to assess the Casorati curvature. This certainly appears to be a “natural” measure, a bit like *size* or (generic) *amount.* As we defined it here, it is simply the root mean square deviation from planarity, a concept that is easily grasped without being aware of any differential geometry. But, differently from the shape index, in this case, there are several contenders. Indeed, the Casorati curvature is hardly ever used in formal differential geometry. According to the task at hand, the mathematician is much more likely to opt for either the Gaussian (or *intrinsic*) curvature, or the “mean” (or *extrinsic*) curvature.^7^ Yet, neither of these has any intuitive appeal to the layman. For instance, the Gaussian curvature is zero for ridges and ruts, whereas the mean curvature is zero for the symmetric saddle: all instances of obviously “curved” surfaces to the naive observer. Moreover, both the Gaussian and the mean curvature are signed quantities, whereas intuitively curvature is a positive magnitude, defined on a ratio scale. Here, the ways of phenomenology and formal geometry evidently have to part.

From bitter experience, we know that it is very hard—in our moments of despair we sometimes would even say “impossible”—to explain the concepts of intrinsic and extrinsic curvature to naive subjects. In contradistinction, showing a few examples suffices to explain Casorati curvature. An explanation of Casorati curvature as a measure of the normal deviations from planarity satisfies the conceptual curiosity of most people that lack a background in differential geometry. From the present data, we see that human observers easily and reliably judge ratios of Casorati curvature. In our case, they did quite well in judging the curvature of instances with respect to a remembered prototype.

**Figure 8.**
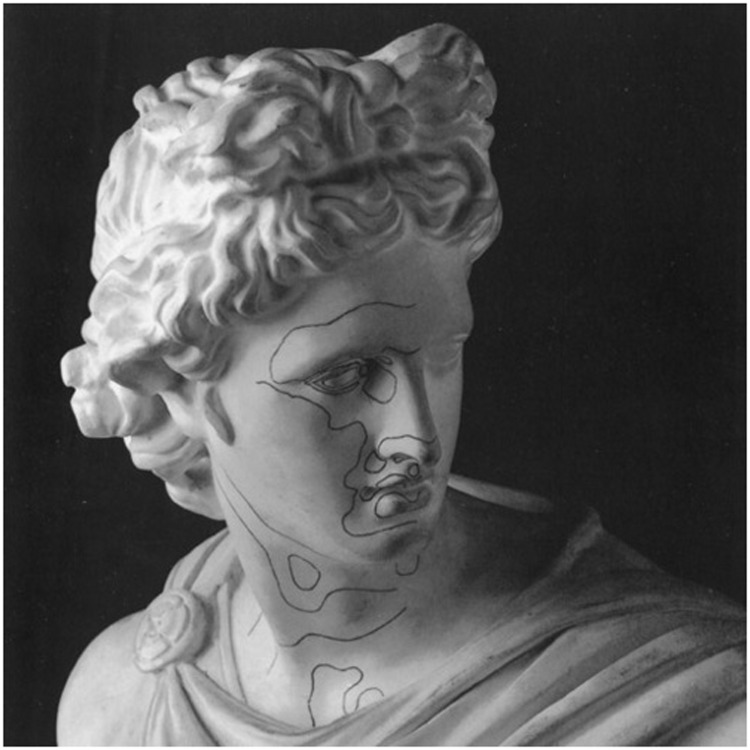
The parabolic curves drawn on a copy bust of the Apollo Belvedere (property of the Mathematics Institute of Göttingen).

Alberti conceived of the local shapes as qualities that were spread over surfaces like paint. That is one reason why we developed the shape index color scale (Koenderink & van Doorn, 1992). One easily imagines a solid body to be painted in these shape index colors. Of course, it remains a matter of experimental phenomenology to explore to what extent observers can actually “see” such distributions. So far, we have collected only scattered observations. Yet, this topic has very attractive potential applications. Well-known is Felix Klein’s speculation that the beauty of human faces is to be sought in the shape of the loci of cups and caps, the yellow and blue isochromes, technically the *parabolic curves*. Felix Klein had a patient student trace these curves on a copy bust of the Apollo Belvedere (of arcane classical beauty!), an item that still survives at the Mathematics Institute of Göttingen (Figure 8). It is not known how the student managed to do this, nor is there any indication that Klein’s hypothesis was supposed to be verified or contradicted (Hilbert & Cohn-Vossen, 1932). Now, more than a century later (!) we are still in no position to assess the value of this surprising brain wave.

## Supplementary Material

Supplementary material

## Notes

1 In cinematographic images of sufficient length, the third dimension is indeed implicit in the stimulus structure. In the minimum case of two successive views, one has the optical flow case (Gibson, 1950), in which the depth dimension is of indefinite scale (Koenderink & van Doorn, 1975, 1991). This also applies to binocular disparity. Thus, binocular disparity and optical flow are cases somewhere between that of full cinematography—or free exploration in the real environment—and (single image) pictorial depth. In neither case does Euclidean differential geometry apply. In this article, the setup is squarely in the ballpark of cinematographic presentation.

2 A good example are the tricks played in the “The Lord of the Rings” productions (director Peter Jackson, 2001/2003, distributed by New Line Cinema). Here, the cinematographic presentation uses ambiguity to the (currently) fullest extent.

3 A “normal direction” only makes sense in isotropic spaces. In pictorial space, one would not know how to even start a definition. Formally, the depth direction itself is normal to anything in pictorial space, but that nullifies its potential application in the differential geometry of pictorial relief (Sachs, 1990; Strubecker, 1962). The tangent plane yields a useful and more natural alternative to the notion of “normal.”

4 Notice that a positive curvature indicates a concave shape and a negative curvature indicates a convex shape. This is the formal convention because the conventional graph of the parabola *y*(*x*)= + *x*^2^ looks like a cup, whereas the graph of the parabola *y*(*x*) = −*x*^2^ looks like a cap.

5 On a smooth surface, the principal curvatures vary smoothly with position. Thus, loci of zero principal curvatures are curves. The planar case occurs at the intersection of such curves, thus isolated points. Isolated points are of measure zero on a twofold extended surface.

6 “Properly defined” is necessary because the integrated square deviation is generically infinite. One needs some device to limit the calculation to an environment of the origin, although the device should not influence the result. This is an easy problem in elementary calculus. The Casorati curvature as defined in the text is

$$\sqrt{\frac{1}{\sigma^{2}}(\int_{-\infty }^{+\infty }\int_{-\infty }^{+\infty }(\frac{k_{1}x^{2}+k_{2}y^{2}}{2}{)}^{2}\frac{e^{-\frac{x^{2}+y^{2}}{2\sigma^{2}}}}{2\pi \sigma^{2}}\text{d}x\text{d}y-(\int_{-\infty }^{+\infty }\int_{-\infty }^{+\infty }(\frac{k_{1}x^{2}+k_{2}y^{2}}{2})\frac{e^{-\frac{x^{2}+y^{2}}{2\sigma^{2}}}}{2\pi \sigma^{2}}\text{d}x\text{d}y{)}^{2})}$$

which is the square root of the standard definition of the variance as the average of the square minus the square of the average: $\text{var}x=\bar{x^{2}}-{\bar{x}}^{2}$. The Gaussian weight $\exp(-(x^{2}+y^{2})/2\sigma^{2})/2\pi \sigma^{2}$ ensures that we look at the local structure. Notice that the width parameter σ falls out of this equation. It does not matter because there are no higher order terms than the second.

7 The Gaussian curvature is best described as the spread of normal directions (a solid angle) for given area (infinitesimal region of interest). Thus, it is a point property of dimension [length]^−2^. The mean curvature is the mean of the normal curvatures over all surface orientations. Thus, it is a rotationally invariant point property of dimension [length]^−1^. One may show that the former is the geometrical, the latter the arithmetic mean of the principal curvatures.
